# Author Correction: Slow light nanocoatings for ultrashort pulse compression

**DOI:** 10.1038/s41467-021-27446-7

**Published:** 2021-11-30

**Authors:** M. Ossiander, Y.-W. Huang, W. T. Chen, Z. Wang, X. Yin, Y. A. Ibrahim, M. Schultze, F. Capasso

**Affiliations:** 1 John A. Paulson School of Engineering and Applied Sciences, Harvard University, 29 Oxford St, Cambridge, MA 02138 USA; 2 Department of Photonics, National Yang Ming Chiao Tung University, Hsinchu, 30010 Taiwan; 3 Institute of Experimental Physics, Graz University of Technology, Petersgasse 16, 8010 Graz, Austria; 4 University of Waterloo, Waterloo, ON N2L 3G1 Canada

**Keywords:** Nanophotonics and plasmonics, Slow light, Ultrafast photonics, Nanophotonics and plasmonics, Slow light, Ultrafast photonics

Correction to: *Nature Communications* 10.1038/s41467-021-26920-6, published online 11 November 2021.

In this article the funding from Office of Naval Research (ONR), under the MURI program, grant no. N00014-20-1-2450, and from the Air Force Office of Scientific Research (AFOSR), under grant no. FA95550-19-1-0135 was omitted. The original article has been corrected.

