# Supplementary material for: Slow light nanocoatings for ultrashort pulse compression
Source: Nat Commun. 2021 Nov 11;12:6518. doi: 10.1038/s41467-021-26920-6 (PMC8586156; doi:10.1038/s41467-021-26920-6)
Supplement: Supplementary file 1 — Supplementary Information [file 41467_2021_26920_MOESM1_ESM.pdf]

Supplementary Information
for

Slow Light Nanocoatings for Ultrashort Pulse Compression

M. Ossiander[1,*], Y.-W. Huang[1,2], W.T. Chen[1], Z. Wang[3], X. Yin[1], Y. A. Ibrahim[1,4], M. Schultze[3], F. Capasso[1,*]

1 John A. Paulson School of Engineering and Applied Sciences, Harvard University, 29 Oxford St, Cambridge, MA 02138, United States
2 Department of Photonics, National Yang Ming Chiao Tung University, Hsinchu 30010, Taiwan.
3 Institute of Experimental Physics, Graz University of Technology, Petersgasse 16, 8010 Graz, Austria
4 University of Waterloo, Waterloo, ON N2L 3G1, Canada
* Corresponding Authors: mossiander@g.harvard.edu, capasso@seas.harvard.edu

Supplementary Figures

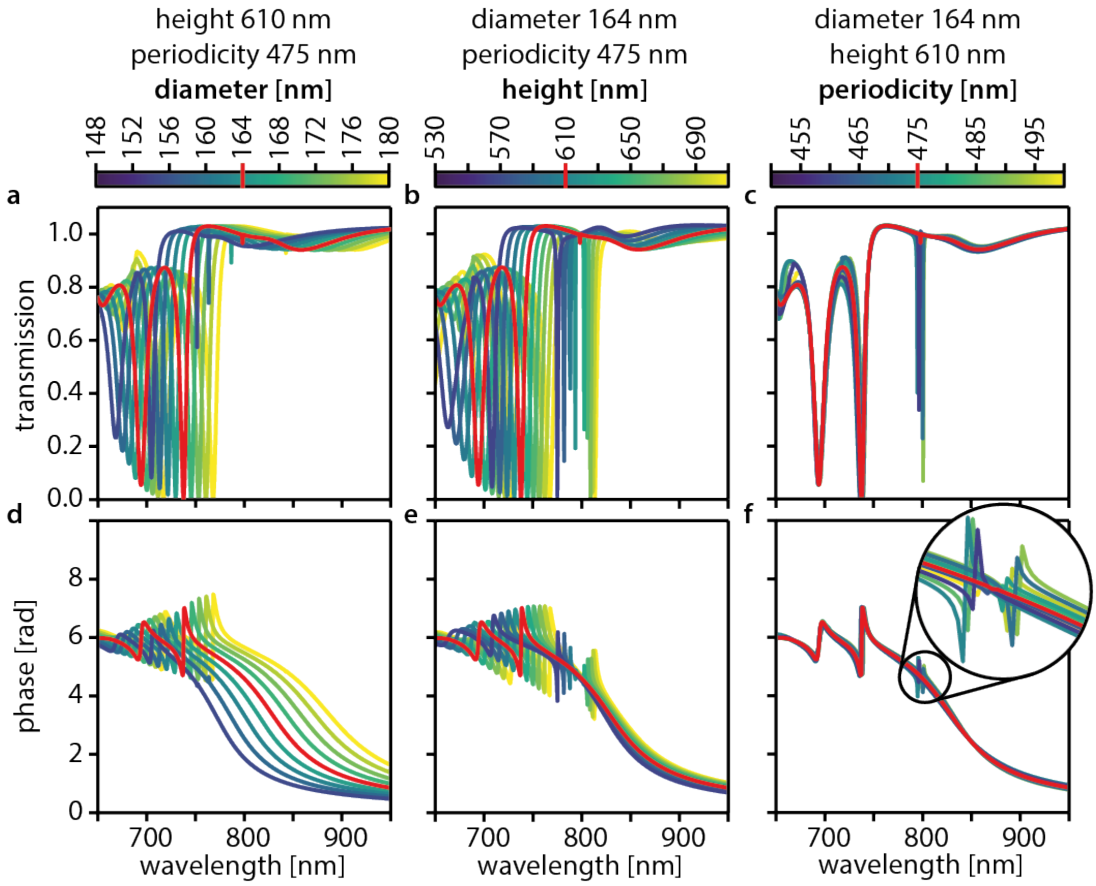

Supplementary Fig. 1

Compressor response to changes in the nanopillar diameter, height, and periodicity. The optimal design for 800 nm central wavelength is marked in red. Compressor transmission characteristics for a) different nanopillar diameters, b) heights, and c) periodicities. Compressor phase profiles for d) different nanopillar diameters, e) heights, and f) periodicities. Panel d) illustrates how the anomalous dispersion region can be tuned by changing the nanopillar diameter. Panels b), c), e), and f) show how the unwanted narrowband resonance at the center of the anomalous dispersion region can be avoided by selecting the correct nanopillar height and periodicity.

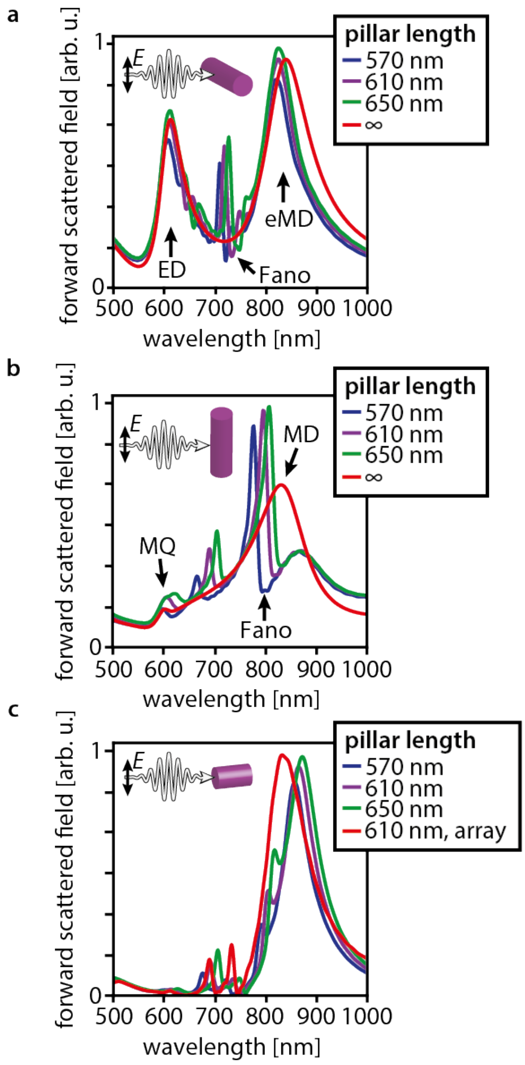

Supplementary Fig. 2

Identification of Mie and Fabry-Perot resonances via forward-scattered field calculations for an individual nanopillar with 164 nm diameter and different nanopillar height. a) forward scattering behavior for light incident perpendicular to the nanopillar with the electric field (*E*) oriented perpendicular to the nanopillar (see inset). For an infinitely long nanopillar (red line), only the Mie-type longitudinal magnetic dipole (eMD) and electric dipole (ED) resonance are visible. For finite nanopillar length, narrow Fabry-Perot-type resonances appear that spectrally shift when changing the nanopillar length. They show a strong Fano-type interaction with the broadband Mie-type resonances. b) forward scattering behavior for light incident perpendicular to the nanopillar with the electric field oriented along the nanopillar. For an infinitely long nanopillar (red line), only the Mie-type broadband transverse magnetic dipole (MD) and magnetic quadrupole (MQ) resonance are visible. For finite nanopillar length, narrowband Fabry-Perot-type resonances appear that also show a strong Fano-type interaction with the broadband Mie-type resonances. c) The Fano-type interaction between the Mie-type and Fabry-Perot-type resonances displayed in panel b) is also visible in the forward scattering behavior of an individual nanopillar when light is incident along the nanopillar axis. Arranging the nanopillars in a 475 nm x 475 nm array (red line) suppresses the interaction.

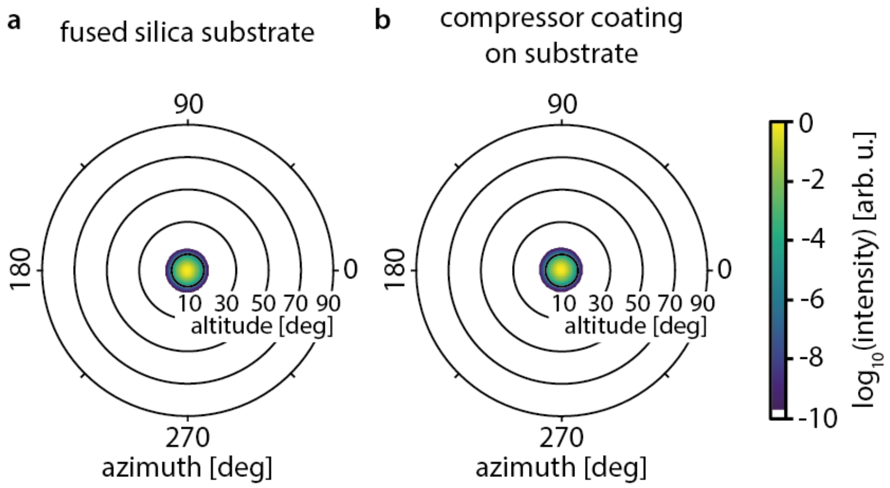

Supplementary Fig. 3

Modeled far-field intensity profile of a Gaussian laser beam (FWHM waist diameter 7 um) in the forward-scattering hemisphere. a) after the beam was transmitted through the fused silica substrate only and b) after the beam was transmitted through the compressor-coated substrate. Because the nanopillar array periodicity is smaller than all wavelengths in the working range in the substrate and in air, the transmitted far-field only contains the zeroth order.

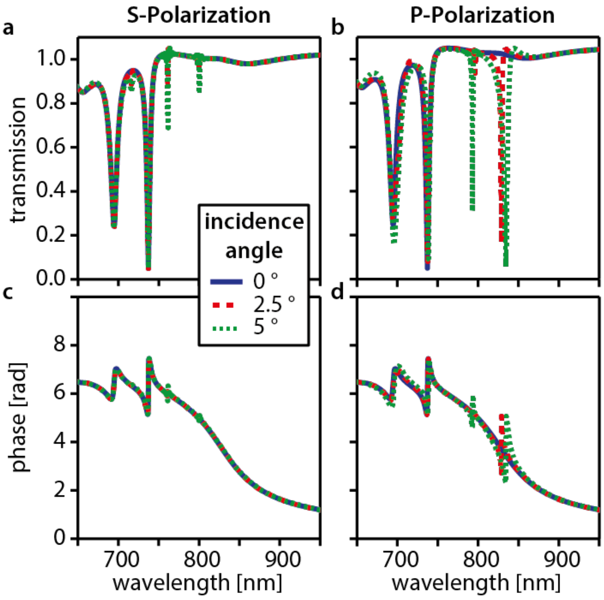

Supplementary Fig. 4

Compressor response (164 nm pillar diameter, 610 nm pillar height, 475 nm periodicity) to different angles of light incidence. Compressor transmission characteristics for a) incident light with s-polarization and b) p-polarization. Compressor phase profiles for c) incident light with s-polarization and d) p-polarization. For s-polarized light, both, the transmission and phase of the compressor are robust against slanted incidence. For p-polarized light, slanted incidence reduces the long-wavelength limit of the working range.

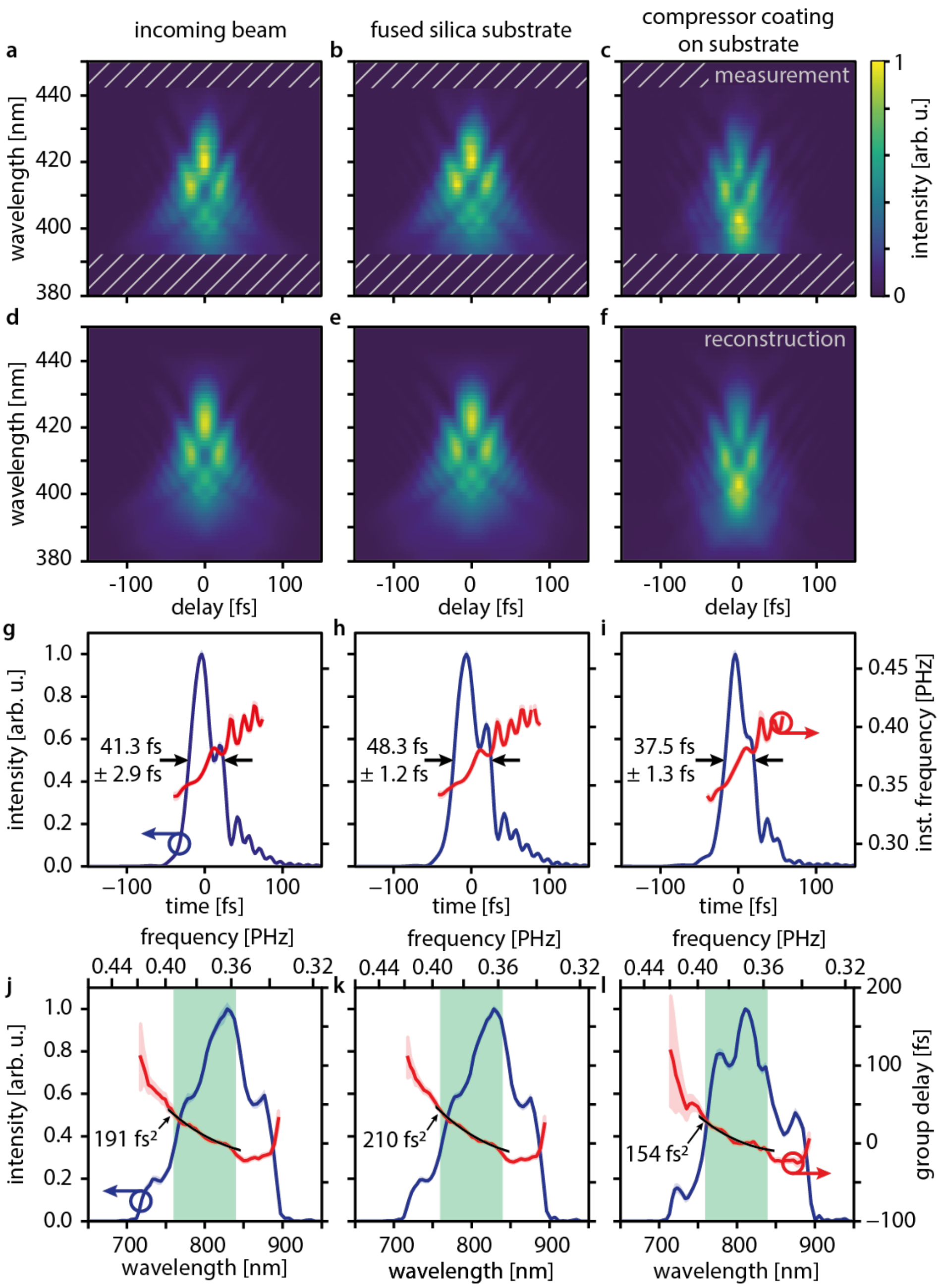

Supplementary Fig. 5.

Compression of ultrashort laser pulses, full data set. a - c) experimental second harmonic frequency resolved optical gating spectrograms (SH-FROG) of the incoming laser pulses (1st column), the pulses after transmission through the compressor substrate only (2nd column), and the pulses after the compressor (3rd column). Hatched areas are not phase-matched and were not used for reconstruction. d-f) spectrograms reconstructed using the iterative ptychographic reconstruction algorithm. g-i) measured time domain intensity (blue lines) and instantaneous frequency (red lines) profiles. Standard deviations were determined using the bootstrap method (blue and red shaded areas). The arrows and labels indicate the pulse full width at half maximum duration. j-l) measured spectral domain intensity (blue lines) and group delay (red lines) profiles and least-squares fits (black lines) to the group delay profiles in the working range (light green). Fits account for 300 fs^3 third order dispersion of the incoming pulses and are labeled with their slope, i.e., the group delay dispersion of the laser pulses.
